# The association between *Matrix Metallo-proteinases-9* (*MMP-9*) gene family polymorphisms and risk of Coronary Artery Disease (CAD): a systematic review and meta-analysis

**DOI:** 10.1186/s12872-020-01510-4

**Published:** 2020-05-19

**Authors:** Reza Hassanzadeh-Makoui, Bahman Razi, Saeed Aslani, Danyal Imani, Seyedeh Samaneh Tabaee

**Affiliations:** 1 Department of Cardiology, School of medicine, Zanjan University of Medical Science (ZUMS), Zanjan, Iran; 2 Department of Hematology and Blood Banking, School of Medicine, Tarbiat modares university (TMU), Tehran, Iran; 3 Department of Immunology, School of medicine, Tehran University of Medical Sciences (TUMS), Tehran, Iran; 4 Department of Immunology, School of Public Health, Tehran University of Medical Sciences (TUMS), Tehran, Iran; 5 Noncommunicable Disease Research Center, Neyshabur University of Medical Science, Imam Khomeini Street, Neyshabur, 9319116911 Iran; 6 Faculty of Medicine, Neyshabur University of Medical Science, Neyshabur, Iran

**Keywords:** Coronary artery disease, Matrix metalloproteinases, Genetic polymorphism, Meta-analysis

## Abstract

**Background:**

We performed a systematic review and meta-analysis of the Matrix metalloproteinases (MMP)-9 (C1562T), MMP-9 (R279Q), MMP-9 (P574R) and MMP-9 (R668Q) polymorphisms and risk of Coronary Artery Disease (CAD).

**Methods:**

After a systematic literature search, pooled odds ratio (OR) and their corresponding 95% confidence interval (CI) were used to evaluate the strength of the association.

**Results:**

We identified 40 studies with 11,792 cases and 8280 controls for C1562T, 7 case-control studies with 5525 cases and 2497 controls for R279Q, 2 studies with 1272 cases and 785 controls for P574R, and 2 studies with 1272 cases and 785 controls for R668Q. MMP-9 (C1562T) polymorphism was associated with increased risk of CAD under dominant model (OR = 1.41, *P* < 0.001), recessive model (OR = 1.59, *P* < 0.001), allelic model (OR = 1.38, *P* < 0.001), TT vs. CC model (OR = 1.70, *P* < 0.001), and CT vs. CC model (OR = 1.35, *P* < 0.001). Moreover, the subgroup analysis based on the continent of the study populations in this SNP indicated strong significant association in Asians but not in Europeans. Subgroup analysis was not performed in Africa, America and Oceania, due to lack of sufficient data.

**Conclusions:**

Our meta-analysis revealed that MMP-9 (C1562T) SNP conferred a susceptibility risk for CAD in the overall analysis and Asian population. The overall analysis and subgroup analysis of the other three SNPs reject the association between MMP-9 polymorphisms and the risk of CAD. Although the results should interpret with caution because of small sample size of included studies in these three SNPs.

## Background

Coronary artery disease (CAD) is a worldwide medical problem that is the leading cause of death in both developed and developing countries, especially in older people [1, 2]. Several studies have shown that the traditional risk factors, such as blood lipid, diabetes, hypertension, obesity play crucial roles in the initiation and perpetuation of CAD. However, it is nowadays accepted that genetic component has an essential role in the development of CAD [3–6]. Researches have suggested that family aggregation of CAD is not unusual, and genetic association investigations revealed that the average heritability of CAD is more than 50% [5, 7]. Epidemiological studies have found many genetic variants especially single-nucleotide polymorphism (SNP) in association with an increased risk of CAD [8]. The exact mechanism underlying the influence of polymorphism on the pathogenesis of CAD is not fully understood. Nevertheless, polymorphisms in numerous genes involved in inflammation, metabolism of lipid and glucose, blood clotting, and homocysteine may affect susceptibility to CAD [9, 10].

. This enzyme is involved in the degradation of extracellular matrix (ECM) components, such as type IV collagen, which is involved in the neovascularization, angiogenesis, inflammatory processes, and development of atherosclerosis.

MMPs are zinc containing enzymes that belong to a neutral protease family. Among the MMP family, MMP-9 is the most important enzyme of this class that is produced by the cells in the vascular wall. Moreover, inflammatory immune cells, such as neutrophils, monocytes as well as endothelial cells and vascular smooth muscle cells (VSMCs) generate MMP-9 [11]. This enzyme is involved in the degradation of extracellular matrix (ECM) components, such as type IV collagen, which is involved in the neovascularization, angiogenesis, inflammatory processes, and development of atherosclerosis [12, 13]. Several studies have shown that the levels of MMPs and their matrix-degrading activity are raised in exposed areas of atherosclerotic plaques, or following acute coronary syndrome [13, 14]. As a result, it is rational to hypothesize that genetic defects resulting in the overexpression of activated MMPs play a crucial role in the pathogenesis of coronary artery disease (CAD).

The MMP family is grouped into gelatinizes (MMP2, 9), collagenases (MMP1, 8, 13, 18), stromelysins (MMP3, 10, 11), and the membrane-type MMPs (MT-MMPs) that are coded by separate genes and have different tissue distribution and bioactive function [15].

Several studies have shown that MMP-9 family polymorphisms might be associated with the risk of CAD [16–18]. However, the results are inconsistent. For example, Mahmoodi et al. conducted a case-control study to investigate the association between -1562C > T genetic polymorphism and susceptibility to CAD. But, genotype and allele frequencies of MMP9 -1562C > T polymorphism were similar between CAD patients and controls (*P* > 0.05) [19]. However, Rodriguez-Perez et al. demonstrated that MMP9 (1562 C > T) allele and the CT genotype were associated with the risk of developing myocardial infraction (MI) [20]. The causes for these controversial results probably due to small sample sizes, different ethnicity, patient selection, clinical heterogeneity, low statistical power, or a combination of these factors. Therefore, we performed this meta-analysis to evaluate whether MMP-9 gene family polymorphisms play a role in CAD susceptibility.

## Methods

We followed a protocol based on observational studies in epidemiology (MOOSE) guidelines [21], and results were reported based on Preferred Reporting Items for Systematic Reviews and Meta-Analyses (PRISMA) guideline [22]. This article does not contain any studies with human participants performed by any of the authors.

### Systematic search strategy

An exhaustive systematic search was conducted through electronic databases (Scopus, Medline) retrieving all potential publications considered the association between MMP-9 family gene polymorphism and susceptibility to CAD. All publications from inception to February 2020 were included (the search was updated before manuscript submission). The combination of key words and Mesh (Medical Subject Headings) terms were as follow: (“matrix metalloproteinase” [Mesh] OR “MMP” OR “gelatinase”) AND (“coronary heart disease” OR “CAD” OR “coronary syndrome” OR “ischemic heart disease” OR “vascular disease” OR “myocardial infarction” OR “MI” OR “atherosclerosis” OR “arteriosclerosis” OR “coronary stenosis” OR “coronary disease” OR “CHD” OR “angina”) AND (“single nucleotide polymorphism” OR “SNP” OR “polymorphisms” OR “mutation” OR “variation”). The references of review articles were cross-checked to find potential publications. Only human studies and English language publications were considered.

### Inclusion and exclusion criteria

We screened retrieval publications according to following inclusion criteria: 1) observational studies (cohort or case-control design); 2) publications considered the association between MMP-9 family gene polymorphism (C1562T, R279Q, P574R and R668Q) and susceptibility to CAD; 3) publications reporting sufficient data to extract or calculate risk estimates with 95% CI; 4) publications reporting numbers or genotype frequencies in cases and healthy controls. Duplicates, book chapters, letters to editor, animal study, case reports, review articles, and studies with repetitive subjects all were excluded. The application of these criteria recognized 40, 7, 2, and 2 eligible studies for C1562T SNP, R279Q SNP, P574R SNP and R668Q SNP, respectively.

### Data extraction

Two authors screened the literature and extracted data independently according to the inclusion and exclusion criteria. The following data was extracted: the first author’s name, journal and year of publication, country of origin, ethnicity, number of subjects in the case and control groups, mean or range of age, genotyping method, genotype counts in the case and control group.

### Quality assessment

The quality of eligible studies was assessed by using the Newcastle-Ottawa Scale (NOS) [23]. Studies were scored based on three main components: selection, comparability, and ascertainment of outcome. This scale ranges from 1 to 9 stare and studies with scores 0–3, 4–6 or 7–9 were of low, moderate, or high-quality, respectively.

### Statistical analysis

In the current meta-analysis, the strengths of association between MMP-9 family gene polymorphism and the risk of CAD was estimated via the OR and 95% CI in five genetic models: dominant model, recessive model, allelic model, homozygote contrast, and heterozygotes contrast. The potential heterogeneity was evaluated by the Q test and the I2 test [23]. According to these two test, if Q had a *P* value less than 0.1 and I2 exceed 50%, the random effects model (REM) was used; otherwise, the fixed effect model (FEM) was applied [24, 25]. Additionally, risk of publication bias was examined by funnel plot, Egger’s weighted regression test and Begg’s rank correlation test (*P* < 0.05 was regarded as statistically significant publication bias) [26, 27]. Besides, quality assessment of genotype data in case control studies was evaluated by Hardy–Weinberg equilibrium (HWE). Finally, in order to show the stability of our results, sensitivity analysis was performed. All statistical tests for this meta-analysis were performed with Stata statistical software (version 14.0; Stata Corporation, College Station, TX, USA) and SPSS (version 23.0; SPSS, Inc. Chicago, IL, USA).

## Results

### Study characteristics

**Fig. 1:**
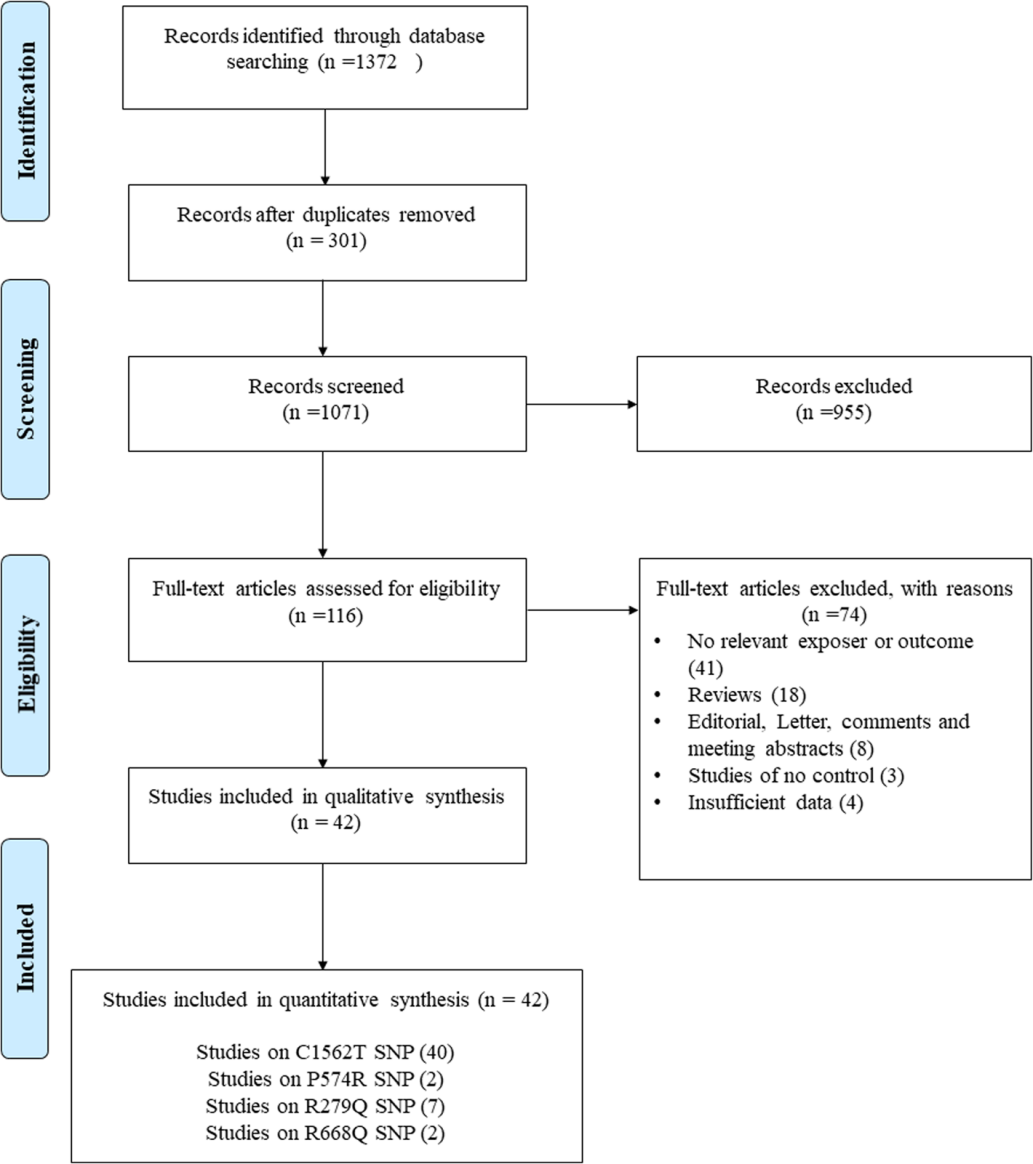
Flow diagram of study selection process

The search and screening process workflow is shown in Fig. 1. Our primary search yielded 1372 records, which 42 of them were included in quantitative analysis [16–20, 28–64]. The studies were published between 2001 to 2019 and all of them had good methodological score ranging between 5 and 8. Polymerase chain reaction-restriction fragment length polymorphism (PCR-RFLP) as genotyping method was common between most of studies. The sample size in case and control groups of four SNPs varied between 40 to 2506 and 40 to 689 individual, respectively. The range of mean ages in case and control groups was from 33 to 94, which means studies were conducted among adults. Only one of included studies had cohort design and the other were case-control. Tables 1 and 2 summarized the characteristics and genotype frequency of the included studies.

**Table 1:** Characteristics of studies included in meta-analysis of overall CAD

Study author	Year	Country	Ethnicity	Study design	Type of CAD	Total cases/controls	Age Case / Control (Mean)	Genotyping method	Quality score
**MMP-9 (C1562T)**									
Pollanen et al.	2001	Finland	European	Case-control	ACS	109 / 167	33–69 / 33–69	PCR-RFLP	6
Wang et al.	2001	Australia	Oceania	Case-control	Stable	619 / 169	57.7 ± 0.5 / NR	PCR-RFLP	7
Cho et al.	2002	Korea	Asian	Case-control	Stable	63 / 134	NR / NR	PCR-RFLP	5
Kim et al.	2002	Korea	Asian	Case-control	Stable	131 / 117	61.3 ± 7.9 / 59.3 ± 8.5	PCR-RFLP	6
Jones et al.	2002	New Zealand	European	Case-control	Stable	414 / 203	71.7 ± 7.6 / 70.8 ± 8.0	PCR-RFLP	8
Tang et al.	2005	China	Asian	Case-control	ACS	101 / 105	NR / NR	PCR-RFLP	5
Chen et al.	2005	China	Asian	Case-control	ACS	78 / 81	NR / NR	PCR-RFLP	5
Meng et al.	2006	China	Asian	Case-control	Stable	117 / 99	NR / NR	PCR-RFLP	5
Nuzzo et al.	2006	Italy	European	Case-control	ACS	49 / 123	NR / NR	PCR-RFLP	5
Chen et al.	2007	China	Asian	Case-control	Stable	150 / 70	NR / NR	PCR-RFLP	5
Nanni et al.	2007	Italy	European	Case-control	ACS	200 / 201	47.8 ± 6.2 / 47.0 ± 5.5	PCR-RFLP	7
Wang et al.	2007	China	Asian	Case-control	ACS	245 / 204	NR / NR	PCR-RFLP	8
Zhang et al.	2008	China	Asian	Case-control	ACS	92 / 95	NR / NR	PCR-RFLP	5
Koh et al.	2008	Korea	Asian	Case-control	ACS	206 / 173	61.1 ± 11.8 / 58.3 ± 11.8	PCR-RFLP	6
Alp et al.	2009	Turkey	European	Case-control	Stable	146 / 122	59.30 ± 9.1 / 57.30 ± 9.7	PCR-RFLP	6
Wu et al.	2009	China	Asian	Case-control	ACS	2517 / 689	NR / 60.42 ± 9.07	PCR-RFLP	8
Gao et al.	2010	China	Asian	Case-control	Stable	96 / 78	NR / NR	PCR-RFLP	5
Fallah et al.	2010	Iran	Asian	Case-control	Stable	145 / 157	58.49 ± 9.12 / 55.35 ± 9.43	PCR-RFLP	6
Yong et al.	2010	China	Asian	Case-control	ACS	128 / 106	NR / NR	PCR-RFLP	5
Ghaderian et al.	2010	Iran	Asian	Case-control	ACS	400 / 200	NR / 65.8 ± 5.9	TaqMan	8
Zhi et al.	2010	China	Asian	Case-control	Stable	762 / 555	67.46 ± 9.61 / 69.90 ± 11.48	PCR-RFLP	8
Wang et al.	2011	China	Asian	Case-control	ACS	384 / 451	55.6 ± 10.9 / 54.1 ± 10.3	PCR-RFLP	8
Opstad et al.	2012	Norway	European	Case-control	Stable	996 / 204	62 / NR	TaqMan	8
Han et al.	2012	China	Asian	Case-control	Stable	91 / 101	NR / NR	PCR-RFLP	5
Saracini et al.	2012	Italy	European	Case-control	Stable	423 / 423	40–94 / 41–94	Nano gene electronic microchip technology	8
Spurthi et al.	2012	India	Asian	Case-control	Stable	100 / 100	56.73 ± 12.2 / 54.55 ± 14.38	PCR-RFLP	5
Sewelam et al.	2013	Egypt	African	Case-control	ACS	40 / 40	NR / NR	PCR-RFLP	5
Wu et al.	2013	China	Asian	Case-control	ACS	258 / 153	63.97 ± 12.32 / 63.61 ± 11.8	PCR-RFLP	7
Xu et al.	2013	China	Asian	Case-control	Stable	382 / 466	62 ± 12 / 62 ± 10	PCR-RFLP	8
Rodriguez et al.	2016	Mexico	American	Case-control	ACS	236 / 285	59 / 58	PCR-RFLP	8
Yin et al.	2016	China	Asian	Case-control	Stable	194 / 251	55.60 ± 10.42 / 56.21 ± 9.83	PCR-RFLP	7
Beton et al.	2016	Turkey	European	Case-control	Stable	200 / 200	60.2 ± 7.4 / 58.3 ± 7.7	PCR-RFLP	7
Daraei et al.	2016	Iran	Asian	Case-control	ACS	117 / 120	62.96 ± 12.80 / 52.55 ± 9.80	PCR-RFLP	6
El-Aziz et al.	2016	Egypt	African	Case-control	ACS	184 / 180	57.2 ± 10.9 / 58.8 ± 8.3	PCR-RFLP	7
Qin et al.	2016	China	Asian	case-control	Stable	261 / 261	58.75 ± 9.36 / 59.21 ± 10.10	PCR-RFLP	7
Peksiene et al.	2017	Lithuania	European	Case-control	ACS	518 / 645	61.9 ± 11.1 / 60.6 ± 11.9	TaqMan	8
Mahmoodi et al.	2017	Iran	Asian	case–control	Stable	100 / 100	59.4 ± 23.5 / 56.7 ± 29.5	PCR-RFLP	5
Xu et al.	2017	China	Asian	Case-control	Stable	264 / 186	59 ± 11.67 / 58 ± 10.72	PCR-RFLP	7
Makrygiannis et al.	2018	Greece	European	Case-control	Stable	175 / 166	72.7 ± 7.6 / 71.5 ± 7.1	PCR-RFLP	7
Malkani et al.	2019	Iran	Asian	Case-control	Stable	101 / 100	59.2 ± 10.2 / 47.3 ± 13.1	PCR-RFLP	5
**MMP-9 (R279Q)**									
Nanni et al.	2007	Italy	European	Case-control	ACS	200 / 201	47.8 ± 6.2 / 47.0 ± 5.5	PCR-RFLP	7
Wu et al.	2009	China	Asian	Case-control	ACS	2506 / 687	NR / 60.42 ± 9.07	PCR-RFLP	8
Zhi et al.	2010	China	Asian	Case-control	Stable	762 / 555	67.46 ± 9.61 / 69.90 ± 11.48	PCR-RFLP	8
Wang et al.	2011	China	Asian	Case-control	ASC	384 / 451	55.6 ± 10.9 / 54.1 ± 10.3	PCR-RFLP	8
Mishra et al.	2012	India	Asian	Cohort	Stable	510 / 230	NR/ 54.2 ± 8.5	PCR-RFLP	8
Opstad et al.	2012	Norway	European	Case-control	Stable	994 / 204	62 / NR	TaqMan	8
Fiotti et al.	2017	Italy	European	Case-control	Stable	169 / 169	69–78 / 67–80	Sequencing	7
**MMP-9 (P574R)**									
Zhi et al.	2010	China	Asian	Case-control	Stable	762 / 555	67.46 ± 9.61 / 69.90 ± 11.48	PCR-RFLP	8
Mishra et al.	2012	India	Asian	Cohort	Stable	510 / 230	NR / 54.2 ± 8.5	PCR-RFLP	8
**MMP-9 (R668Q)**									
Zhi et al.	2010	China	Asian	Case-control	Stable	762 / 555	67.46 ± 9.61 / 69.90 ± 11.48	PCR–RFLP	8
Mishra et al.	2012	India	Asian	Cohort	Stable	510 / 230	NR / 54.2 ± 8.5	PCR–RFLP	8

NR, not reported; ACS, Acute coronary syndrome

**Table 2:** Distribution of genotype and allele among CAD patients and controls

**Study author**	**CAD cases**					**Healthy control**					**P-HWE**	**MAF**
	**CC**	**CT**	**TT**	**C**	**T**	**CC**	**CT**	**TT**	**C**	**T**		
**MMP-9 (C1562T)**												
Pollanen et al.	78	21	10	177	41	124	30	13	278	56	0	0/168
Wang et al.	479	128	12	1086	152	128	41	0	297	41	0/072	0/121
Cho et al.	48	15	0	111	15	67	63	4	197	71	0/016	0/265
Kim et al.	99	32	0	230	32	85	32	0	202	32	0/086	0/137
Jones et al.	257	145	12	659	169	145	57	1	347	59	0/063	0/145
Tang et al.	73	27	1	173	29	91	13	1	195	15	0/494	0/071
Chen et al.	57	21	0	135	21	73	8	0	154	8	0/640	0/049
Meng et al.	91	26	0	208	26	80	18	1	178	20	0/991	0/101
Nuzzo et al.	7	39	3	53	45	86	36	1	208	38	0/181	0/154
Chen et al.	97	48	5	242	58	61	6	3	128	12	0	0/086
Nanni et al.	136	62	2	334	66	135	63	3	333	69	0/147	0/172
Wang et al.	191	52	2	434	56	178	25	1	381	27	0/903	0/066
Zhang et al.	67	22	3	156	28	83	12	0	178	12	0/511	0/063
Koh et al.	151	52	3	354	58	142	31	0	315	31	0/195	0/090
Alp et al.	99	42	5	240	52	90	29	3	209	35	0/718	0/143
Wu et al.	1995	495	27	4485	549	545	143	1	1233	145	0	0/105
Gao et al.	49	38	9	136	56	59	18	1	136	20	0/775	0/128
Fallah et al.	11	57	77	79	211	19	76	62	114	200	0/558	0/637
Yong et al.	97	30	1	224	32	92	14	0	198	14	0/466	0/066
Ghaderian et al.	296	88	16	680	120	141	53	6	335	65	0/708	0/163
Zhi et al.	585	174	3	1344	180	442	110	3	994	116	0/164	0/105
Wang et al.	286	87	11	659	109	373	72	6	818	84	0/244	0/093
Opstad et al.	756	225	15	1737	255	154	46	4	354	54	0/794	0/132
Han et al.	65	25	1	155	27	75	25	1	175	27	0/489	0/134
Saracini et al.	313	98	12	724	122	307	101	15	715	131	0/071	0/155
Spurthi et al.	40	47	13	127	73	48	46	6	142	58	0/241	0/290
Sewelam et al.	32	7	1	71	9	40	0	0	80	0	0	0
Wu et al.	193	56	9	442	74	131	22	0	284	22	0/337	0/072
Xu et al.	268	109	5	645	119	361	103	2	825	107	0/059	0/115
Rodriguez et al.	210	26	0	446	26	271	14	0	556	14	0/670	0/025
Yin et al.	98	73	23	269	119	157	84	10	398	104	0/766	0/207
Beton et al.	158	38	4	354	46	154	43	3	351	49	0/999	0/123
Daraei et al.	66	50	1	182	52	79	38	3	196	44	0/528	0/183
El-Aziz et al.	125	52	7	302	66	141	36	3	318	42	0/690	0/117
Qin et al.	134	100	27	368	154	171	85	5	427	95	0/129	0/182
Peksiene et al.	340	156	22	836	200	431	185	29	1047	243	0/115	0/188
Mahmoodi et al.	68	27	5	163	37	72	26	2	170	30	0/844	0/150
Xu et al.	188	69	7	445	83	151	31	4	333	39	0/126	0/105
Makrygiannis et al.	133	40	2	306	44	133	31	2	297	35	0/898	0/105
Malkani et al.	79	3	19	161	41	100	0	0	200	0	0	0
**Study author**	**CAD cases**					**Healthy control**					**P-HWE**	**MAF**
	**AA**	**AG**	**GG**	**A**	**G**	**AA**	**AG**	**GG**	**A**	**G**		
**MMP-9 (R279Q)**												
Nanni et al.	85	94	21	264	136	94	87	20	275	127	0/984	0/316
Wu et al.	1177	1102	227	3456	1556	297	312	78	906	468	0/772	0/341
Zhi et al.	398	296	68	1092	432	267	226	62	760	350	0/179	0/315
Wang et al.	185	150	49	520	248	239	167	45	645	257	0/052	0/285
Mishra et al.	114	253	143	481	539	53	103	74	209	251	0/142	0/546
Opstad et al.	405	472	117	1282	706	79	98	27	256	152	0/693	0/373
Fiotti et al.	75	69	25	219	119	57	88	24	202	136	0/282	0/402
**Study author**	**CAD cases**					**Healthy control**					**P-HWE**	**MAF**
	**PP**	**PR**	**RR**	**P**	**R**	**PP**	**PR**	**RR**	**P**	**R**		
**MMP-9 (P574R)**												
Zhi et al.	406	296	60	1108	416	279	231	45	789	321	0/770	0/155
Mishra et al.	346	150	14	842	178	169	57	4	395	65	0/747	0/276
**Study author**	**CAD cases**					**Healthy control**					**P-HWE**	**MAF**
	**RR**	**RQ**	**QQ**	**R**	**Q**	**RR**	**RQ**	**QQ**	**R**	**Q**		
**MMP-9 (R668Q)**												
Zhi et al.	564	179	19	1307	217	398	141	16	937	173	0/416	0/289
Mishra et al.	191	286	33	668	352	113	107	10	333	127	0/012	0/141

*P-HWE p*-value for Hardy–Weinberg equilibrium, *MAF* minor allele frequency of control group

### Meta-analysis of MMP-9 (C1562T) and risk of CAD

**Fig. 2:**
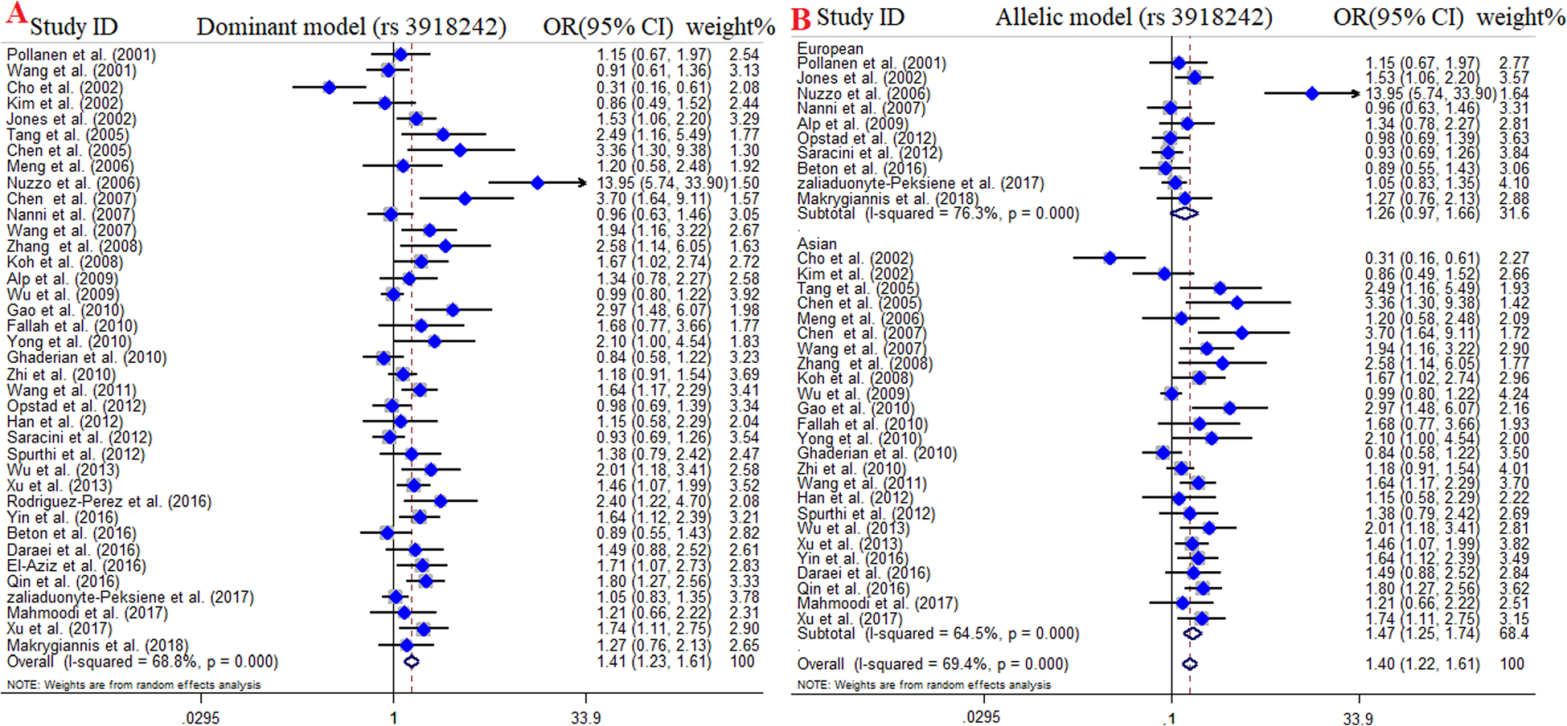
Pooled odds OR and 95% confidence interval of individual studies and pooled data for the association between MMP-9 (C1562T) polymorphism and the risk of CAD in overall populations. **a** Dominat model, **b** Allelic model

A total of 40 studies with 11,792 cases and 8280 controls were included in quantitative synthesis of the association between MMP-9 (C1562T) polymorphism and CAD susceptibility [16–20, 28–49, 51–61, 63, 64]. Among included studies, 26 studies were carried out in Asian countries, 10 studies were in European countries, 2 studies were in African countries, one study in America, and one in Oceania. Since there were only two studies for Africans and one study for American and Oceania, we excluded them from subgroup analysis. The pooled OR divulged a strong positive association between MMP-9 (C1562T) polymorphism and risk of CAD and announced this SNP as a risk factor for CAD. In details, dominant model (OR = 1.41, 95% CI = 1.23–1.61, *P* < 0.001), recessive model (OR = 1.59, 95% CI = 1.29–1.96, *P* < 0.001), allelic model (OR = 1.38, 95% CI = 1.23–1.55, *P* < 0.001), TT vs. CC model (OR = 1.70, 95% CI = 1.35–2.13, *P* < 0.001), and CT vs. CC model (OR = 1.35, 95% CI = 1.18–1.54, *P* < 0.001). FEM was used for recessive and homozygote compressions and REM was applied for dominant, heterozygote, and allelic models. Furthermore, the results of subgroup analysis by ethnicity remarkably showed that MMP-9 (C1562T) polymorphism increase the susceptibility of CAD in the Asian under all genotyping models; dominant model (OR = 1.47, 95% CI = 1.25–1.74, *P* < 0.001), recessive model (OR = 2.06, 95% CI = 1.57–2.71, *P* < 0.001), allelic model (OR = 1.45, 95% CI = 1.26–1.67, *P* < 0.001), TT vs. CC model (OR = 2.42, 95% CI = 1.77–3.32, *P* < 0.001), and CT vs. CC model (OR = 1.39, 95% CI = 1.19–1.64, *P* < 0.001) (Fig. 2). No statistically significant association was observed in Europeans.

The stratification of studies also performed based on type of CAD, including acute coronary syndrome (ACS) and stable angina. The findings demonstrated a statistically significant association between *MMP9* (C1562T) polymorphism and stable angina susceptibility across all genotype model. However, the positive association between MMP-9 (C1562T) polymorphism and ACS susceptibility was observed in dominant model (OR = 1.66, 95% CI = 1.32–2.10, *P* < 0.001, REM), allelic model (OR = 1.57, 95% CI = 1.29–1.92, *P* < 0.001, REM), CT vs. CC model (OR = 1.62, 95% CI = 1.28–2.04, *P* < 0.001, REM), but not recessive model (OR = 1.32, 95% CI = 0.93–1.86, *P* = 0.12, FEM) and TT vs. CC model (OR = 1.40, 95% CI = 0.99–1.98, *P* = 0.06. The results of pooled ORs, heterogeneity tests and publication bias tests in different analysis models are shown in Table 3.

**Table 3:** Main results of pooled ORs in meta-analysis of *MMP9* gene polymorphisms and CAD risk

Subgroup		Sample size	Test of association		Test of heterogeneity		Test of publication bias (Begg’s test)		Test of publication bias (Egger’s test)	
	Genetic model	Case/Control	OR	95% CI (***p***-value)	I^**2**^ (%)	P	Z	P	T	P
**MMP-9 (C1562T)**										
**Overall**	Dominant model	11,792 / 8280	**1.41**	**1.23–161 (≤0.001)**	**68.8**	**≤0.001**	1.48	0.13	1.62	0.11
	Recessive model	11,792 / 8280	**1.59**	**1.29–1.96 (≤0.001)**	**18.3**	**0.19**	1.34	0.17	1.49	0.14
	Allelic model	11,792 / 8280	**1.38**	**1.23–1.55(≤0.001)**	**68.7**	**≤0.001**	1.39	0.16	1.54	0.13
	TT vs. CC	11,792 / 8280	**1.70**	**1.35–2.13 (≤0.001)**	**34**	**0.42**	1.56	0.11	2.05	0.04
	CT vs. CC	11,792 / 8280	**1.35**	**1.18–1.54 (≤0.001)**	**65.4**	**≤0.001**	1.48	0.13	1.62	0.11
**Subgroup**										
**Asian**	Dominant model	7483 / 5152	**1.47**	**1.25–1.74 (≤0.001)**	64.5	≤0.001	−0.72	0.47	−0.14	0.88
	Recessive model	7483 / 5152	**2.06**	**1.57–2.71 (≤0.001)**	0	0.45	1.94	0.05	1.39	0.18
	Allelic model	7483 / 5152	**1.45**	**1.26–1.67 (≤0.001)**	64.3	≤0.001	0.99	0.32	1.64	0.15
	TT vs. CC	7483 / 5152	**2.42**	**1.77–3.32 (≤0.001)**	0	0.45	0	1	−0.18	0.86
	CT vs. CC	7483 / 5152	**1.39**	**1.19–1.64(≤0.001)**	60.8	≤0.001	−0.25	0.80	0.15	0.88
**European**	Dominant model	3230 / 2331	1.26	0.97–1.66 (0.08)	76.3	≤0.001	0.78	0.45	0.84	0.43
	Recessive model	3230 / 2331	1.05	0.75–1.47 (0.77)	0	0.59	1.04	0.29	0.62	0.55
	Allelic model	3230 / 2331	1.22	0.97–1.53(0.08)	75.5	≤0.001	−1.73	0.08	−0.69	0.51
	TT vs. CC	3230 / 2331	1.10	0.78–1.54 (0.59)	32.1	0.15	−0.21	0.83	0.2	0.82
	CT vs. CC	3230 / 2331	1.25	0.96–1.64 (0.09)	74.1	≤0.001	−0.25	0.80	−0.68	0.52
**ACS**	Dominant model	5862 / 4018	**1.66**	**1.32–2.10 (≤0.001)**	76.1	≤0.001	−0.25	0.80	0.89	0.39
	Recessive model	5862 / 4018	1.32	0.93–1.86 (0.12)	2.8	0.416	−0.25	0.80	−0.63	0.54
	Allelic model	5862 / 4018	**1.57**	**1.29–1.92 (≤0.001)**	74.5	≤0.001	−0.35	0.72	−0.75	0.46
	TT vs. CC	5862 / 4018	1.40	0.99–1.98 (0.06)	35.1	0.11	0.05	0.96	−0.57	0.57
	CT vs. CC	5862 / 4018	**1.62**	**1.28–2.04 (≤0.001)**	75	≤0.001	−0.45	0.65	−0.99	0.34
**Stable**	Dominant model	5930 / 4262	**1.26**	**1.07–1.48 (≤0.001)**	60.9	≤0.001	0.38	0.70	0.24	0.81
	Recessive model	5930 / 4262	**1.77**	**1.37–2.30 (≤0.001)**	23.6	0.18	−0.12	0.90	−0.42	0.68
	Allelic model	5930 / 4262	**1.26**	**1.09–1.46 (≤0.001)**	63.9	≤0.001	0.12	0.90	−0.49	0.63
	TT vs. CC	5930 / 4262	**1.95**	**1.45–2.64 (≤0.001)**	31.9	0.10	1.57	0.11	14.14	0.04
	CT vs. CC	5930 / 4262	**1.20**	**1.03–1.39 (0.01)**	52.2	≤0.001	0.52	0.60	0.38	0.76
**MMP-9 (R279Q)**										
**Overall**	Dominant model	5525 / 2497	0.92	0.83–1.02 (0.12)	38.7	0.13	0.05	0.96	−0.23	0.83
	Recessive model	5525 / 2497	0.88	0.76–1.02 (0.08)	0	0.48	−0.18	0.85	−0.21	0. 83
	Allelic model	5525 / 2497	0.93	0.86–1(0.05)	38.1	0.13	0.05	0.96	−0.06	0.95
	GG vs. AA	5525 / 2497	0.86	0.73–1.01(0.07)	17.9	0.29	0.45	0.65	0.33	0.74
	AG vs. AA	5525 / 2497	0.94	0.85–1.05 (0.26)	29.7	0.20	0.19	0.85	−0.19	0.85
**Subgroup**										
**Asian**	Dominant model	4162/ 1923	0.93	0.83–1.04 (0.19)	45.4	0.13	−0.98	0.32	−1.70	0.18
	Recessive model	4162/ 1923	0.86	0.72–1.01 (0.06)	36	0.19	0.56	0.57	0.37	0.73
	Allelic model	4162/ 1923	0.92	0.85–1 (0.06)	59.6	0.06	0.09	0.92	−0.03	0.97
	GG vs. AA	4162/ 1923	0.85	0.71–1.02 (0.08)	53.7	0.09	1.16	0.24	0.92	0.38
	AG vs. AA	4162/ 1923	0.95	0.84–1.07 (0.41)	15.3	0.31	1.34	0.18	1.58	0.15
**European**	Dominant model	1363 / 574	0.91	0.74–1.13 (0.38)	53.2	0.11	0.27	0.78	0.46	0.65
	Recessive model	1363 / 574	0.96	0.70–1.32 (0.80)	0	0.84	0.55	0.58	0.74	0.47
	Allelic model	1363 / 574	0.94	0.81–1.10 (0.45)	10.1	0.32	0.27	0.78	0.10	0.92
	GG vs. AA	1363 / 574	0.90	0.64–1.26 (0.53)	0	0.68	0	1	0.38	0.71
	AG vs. AA	1363 / 574	0.91	0.73–1.14 (0.39)	58.9	0.08	0.52	0.60	−0.47	0.72
**MMP-9 (P574R)**										
**Overall**	Dominant model	1272 / 785	1.05	0.72–1.53 (0.81)	0.69	0.07	*	*	*	*
	Recessive model	1272 / 785	1.01	0.69–1.49 (0.95)	0	0.47	*	*	*	*
	Allelic model	1272 / 785	0.93	0.79–1.10 (0.41)	0	0.41	*	*	*	*
	RR vs. PP	1272 / 785	0.97	0.55–1.44 (0.87)	0	0.38	*	*	*	*
	PR vs. PP	1272 / 785	1.03	0.72–1.48 (0.87)	63.7	0.09	*	*	*	*
**MMP-9 (R668Q)**										
**Overall**	Dominant model	1272 / 785	1.19	0.66–2.13 (0.56)	88.3	≤0.001	*	*	*	*
	Recessive model	1272 / 785	1.12	0.68–1.84 (0.64)	21.2	0.26	*	*	*	*
	Allelic model	1272 / 785	1.11	0.73–1.69 (0.62)	85.1	0.01	*	*	*	*
	QQ vs. RR	1272 / 785	1.26	0.55–2.89 (0.58)	63.1	0.01	*	*	*	*
	RQ vs. RR	1272 / 785	1.18	0.68–2.06 (0.43)	86.4	≤0.001	*	*	*	*

*Begg’s and Egger’s test were not calculable

*ACS* acute coronary syndrome, *OR* odds ratio, *CI* confidence interval, *MMP* matrix metalloproteinase

### Meta-analysis of MMP-9 (R279Q) and risk of CAD

**Fig. 3:**
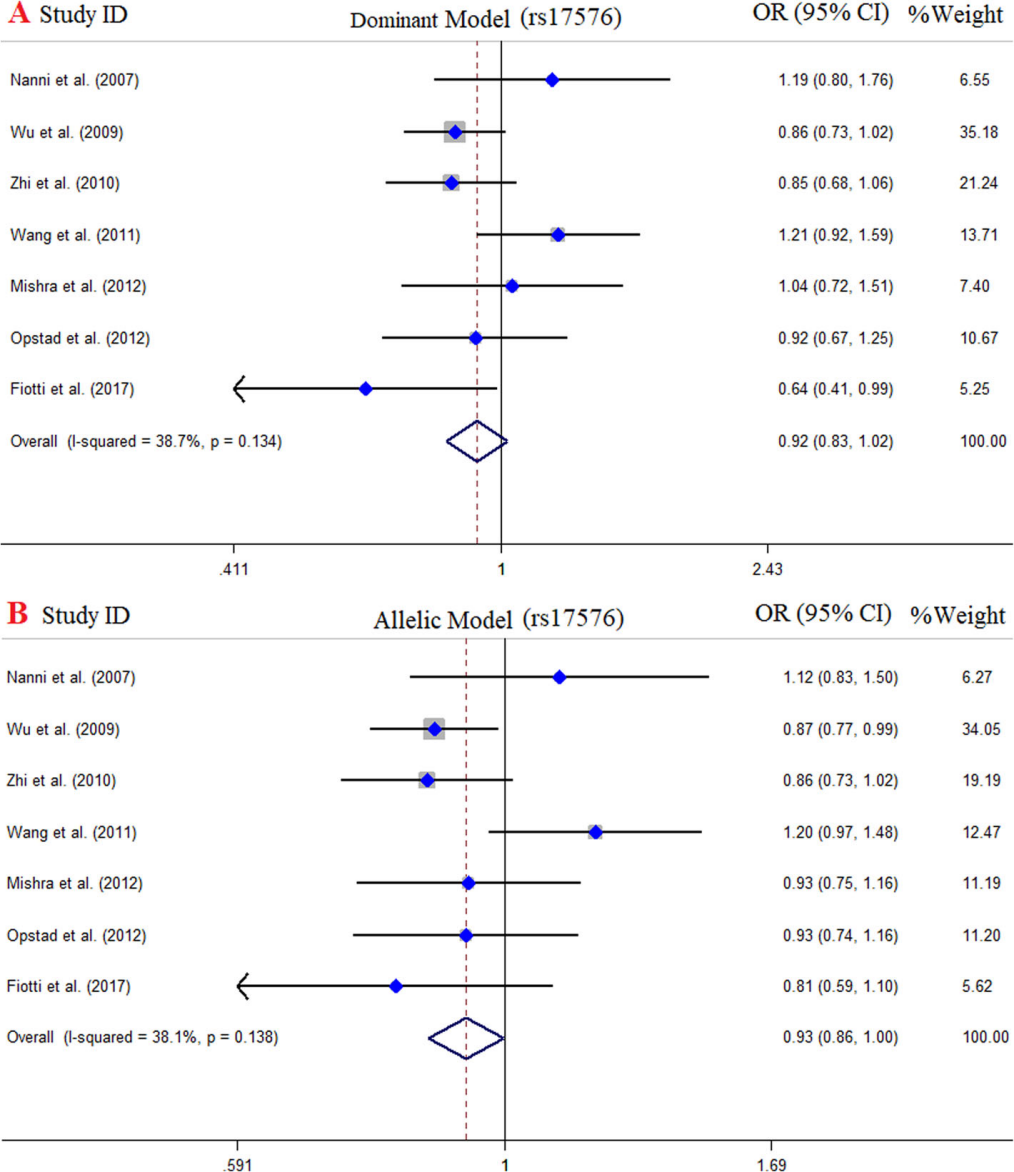
Pooled odds OR and 95% confidence interval of individual studies and pooled data for the association between MMP-9 (R279Q) polymorphism and the risk of CAD in overall populations. **a** Dominant model, **b** Allelic model

There were 7 case-control studies with 5525 cases and 2497 controls concerning MMP-9 (R279Q) polymorphism and risk of CAD [18, 38, 42, 47, 48, 50, 62]. Of those, 4 studies were performed in Asians and 3 studies were in Europeans. The pooled results indicated a negative, but not significant, association between MMP-9 (R279Q) gene polymorphism and CAD risk under all genotype models for the overall population and subgroup analysis (Fig. 3). The results of pooled ORs, heterogeneity tests and publication bias tests in different analysis models are shown in Table 3.

### Meta-analysis of MMP-9 (P574R) and risk of CAD

For MMP-9 (P574R) SNP, two studies with 1272 case and 785 controls were included for quantitative analysis [47, 50]. Studies were carried out in China and India. The results of overall population reject any association between MMP-9 (P574R) SNP and risk of CAD across all genotype models. The results of pooled ORs, heterogeneity tests and publication bias tests in different analysis models are shown in Table 3.

### Meta-analysis of MMP-9 (R668Q) and risk of CAD

Two studies with 1272 case and 785 controls were included for quantitative analysis for MMP-9 (R668Q) SNP [47, 50]. Studies were carried out in China and India. There was no evidence of significant association between MMP-9 (R668Q) SNP and risk of CAD under all genotype models. The results of pooled ORs, heterogeneity tests and publication bias tests in different analysis models are shown in Table 3.

### Publication bias and heterogeneity

**Fig. 4:**
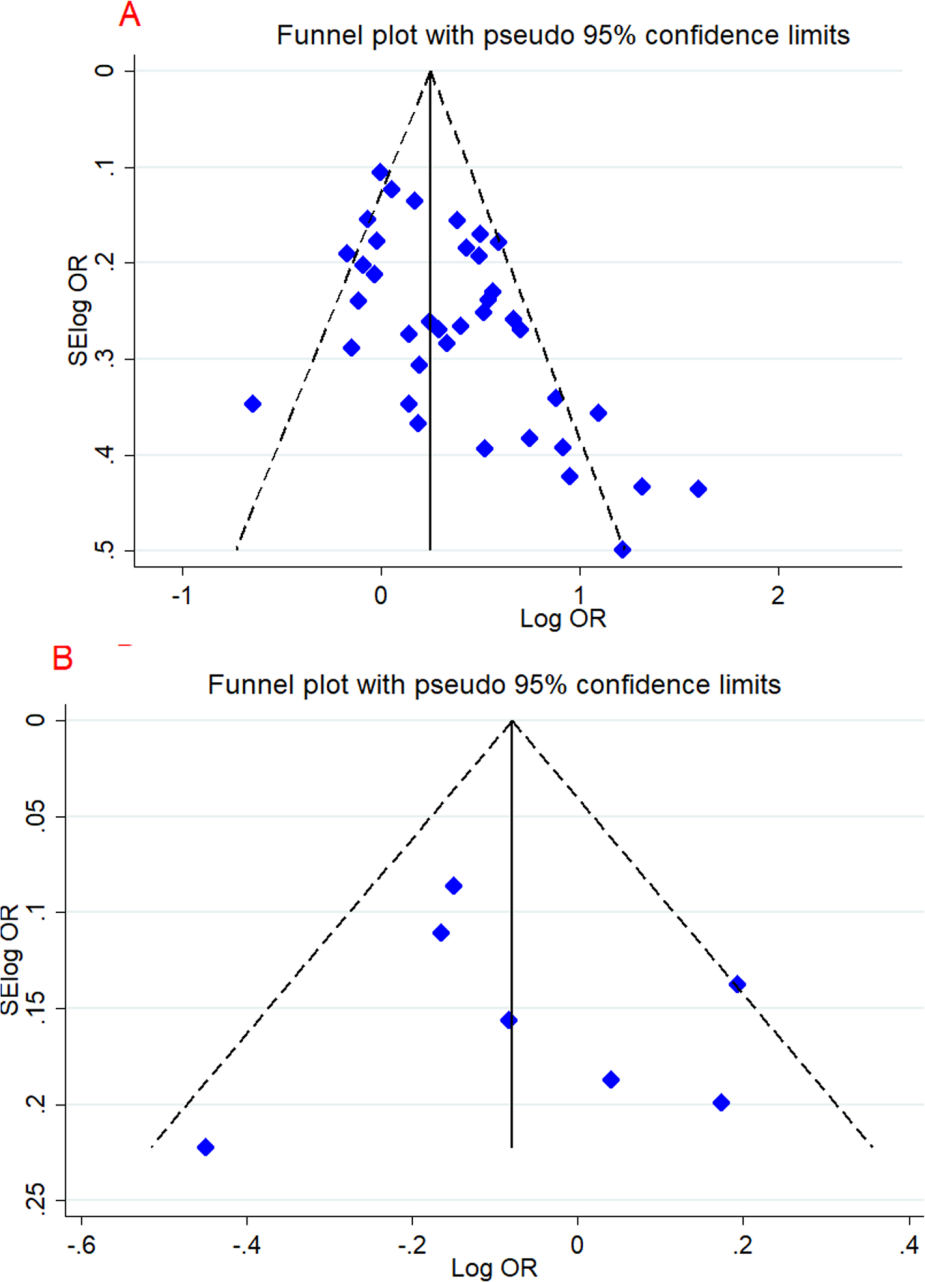
Begg’s funnel plot for publication bias test. Each point represents a separate study for the indicated association. **a** Dominant model (C1562T), **b** Dominant model (R279Q)

In this study, we used Egger’s regression test, Begg’s adjusted rank correlation test and visual examination of the funnel plot (just for C1562T and R279Q) to measure publication bias (Fig. 4). Overall, no significant publication bias was detected. Besides, the estimation of heterogeneity by I^2^ and Q test was significant in some models (Table 3).

### Sensitivity analysis

**Fig. 5:**
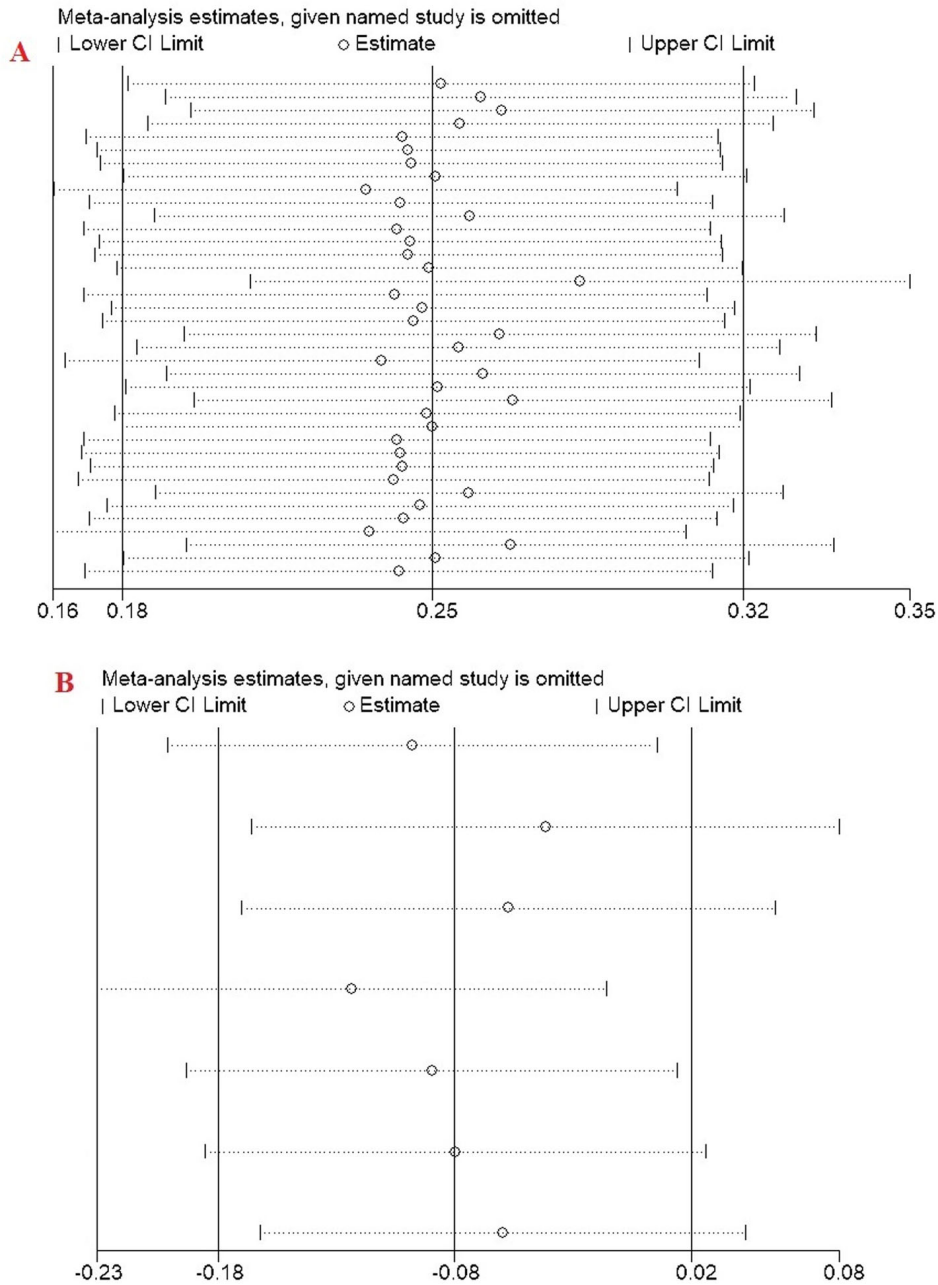
Sensitivity analysis in present meta-analysis estimates the individual influence of studies on pooled results. **a** Dominant model (rs C1562T), **b** Dominant model (R279Q)

The leave-one-out method was used in the sensitivity analysis to explore the effect of individual data on the pooled ORs (just for C1562T and R279Q). The significance of ORs was not altered through omitting any single study, indicating that our results were statistically robust **(**Fig. 5).

## Discussion

Up until now, numerous investigations have been performed to address the association between SNPs of the MMP gene family, including MMP-9 (C1562T), MMP-9 (R279Q), MMP-9 (P574R), and MMP-9 (R668Q) polymorphisms and risk of CAD. The findings of these investigations have sometimes been in accordance with each other, but sometimes conflicting. By meeting the limitations of the individual studies, such as little statistical strength and small sample size, meta-analysis studies provide a beneficial tool to settle these limitations and confer a conclusive outcome. In order to solve this issue in respective of MMP-9 gene family polymorphisms and risk of CAD, here we carried out this meta-analysis by including the most comprehensive and up-to-date original studies worldwide to come up with valid approximation of this association. In the current meta-analysis, we included 40 studies with 11,792 cases and 8280 controls for MMP-9 (C1562T) SNP, 7 case-control studies with 5525 cases and 2497 controls for MMP-9 (R279Q) SNP, 2 studies with 1272 case and 785 controls for MMP-9 (P574R) SNP, and 2 studies with 1272 case and 785 controls for MMP-9 (R668Q) SNP. The analysis indicated that MMP-9 (C1562T) SNP was significantly associated with increased risk of CAD susceptibility in the overall analysis and Asian population particularly.

Different human and animal experiments have suggested that there is an elevated level of MMP-9 in the atherosclerotic arteries in comparison to health controls. It was also shown that MMP-9 is predominantly active in the lipid core margin of the atherosclerotic plaques, the shoulder regions of plaque, and in regions with active formation of microvessels. As a consequence, it appears that MMP-9 plays a critical role in the stability/instability of the coronary artery plaques and development of myocardial infarction during CAD [65]. In addition, researchers have observed that in mice knock-out for the *MMP9* gene, migration potency of the VSMCs as well as atherosclerosis lesions were reduced in comparison to the wild-type animals [66]. According to clinical observations, upregulation of MMP-9 exhibited a correlation with instability of the atherosclerosis plaque and premature CAD development [67]. Based on the prospective studies, serum levels of MMP-9 could confer a tool to estimate the mortality risk during the cardiovascular diseases [68].

Zhang et al. in the 214 meta-analysis, by including 26 studies containing 12,776 cases and 6371 controls, indicated that MMP-9 (C1562T) polymorphism was not associated with the risk of CAD in the overall results [69]. However, they reported that MMP-9 (C1562T) SNP is involved in the decrease susceptibility to CAD in Asian population. In 2016, a meta-analysis was conducted on 10 case-control studies to assess the possible relationship between the MMP-9 (C1562T) SNP and CAD in the Chinese Han population. This study indicated that all genetic comparisons of the MMP-9 (C1562T) SNP increased the risk of CAD in the Chinese Han population [70]. In the current meta-analysis, association between SNPs of the MMP-9 gene family, including MMP-9 (C1562T), MMP-9 (R279Q), MMP-9 (P574R), and MMP-9 (R668Q) polymorphisms and risk of CAD was evaluated. Our literature search led to identification and inclusion of 40 studies with 11,792 cases and 8280 controls for MMP-9 (C1562T) SNP, 7 case-control studies with 5525 cases and 2497 controls for MMP-9 (R279Q) SNP, 2 studies with 1272 case and 785 controls for MMP-9 (P574R) SNP, and 2 studies with 1272 case and 785 controls for MMP-9 (R668Q) SNP. Therefore, this is the most comprehensive meta-analysis of MMP-9 gene family polymorphisms and risk of CAD to date (March 2020). Our analysis revealed that MMP-9 (C1562T) polymorphism increased the risk of CAD in the overall analysis under dominant (OR = 1.41), recessive (OR = 1.59), allelic (OR = 1.38), homozygous TT vs. CC (OR = 1.70), and heterozygous CT vs. CC (OR = 1.35) models. In contrast to Zhang et al. [69] study, we noticed that MMP-9 (C1562T) polymorphism increased the susceptibility of CAD risk in the Asian population under all genotyping models; dominant (OR = 1.47), recessive (OR = 2.06), allelic (OR = 1.45), homozygous TT vs. CC (OR = 2.42), and heterozygous CT vs. CC (OR = 1.39) models. However, other three polymorphisms of the *MM9* gene, including MMP-9 (R279Q), MMP-9 (P574R), and MMP-9 (R668Q) polymorphisms, were not associated with CAD risk.

Regulatory mechanisms at the transcriptional level is involved in the modulation of MMP-9 expression. The MMP-9 (C1562T) SNP is harbored within the 9 bp sequence GCGCAC/TGCC (− 1567 → − 1559), which is considered as a regulatory element of the gene and confers a site for binding of molecules involved in the inhibition of transcription [71]. It was found that an alteration in the binding site structure by substitution of the MMP-9-1562 C allele with − 1562 T allele led to decreased binding potential of the proteins involved in the inhibition of transcription to the DNA sequence [30]. As a result, MMP-9 (C1562T) SNP plays a role in orchestrating the transcription activity of MMP-9 and, hence, modulate the susceptibility risk to several diseases. Therefore, we analyzed the available data to gain a wide understanding of this SNP in case of CAD. We noticed that T allele representation was increased in all models of MMP-9 (C1562T) SNP comparison in the overall analysis, including dominant model (OR = 1.41, 95% CI = 1.23–1.61, *P* < 0.001), recessive model (OR = 1.59, 95% CI = 1.29–1.96, *P* < 0.001), allelic model (OR = 1.38, 95% CI = 1.23–1.55, *P* < 0.001), TT vs. CC model (OR = 1.70, 95% CI = 1.35–2.13, *P* < 0.001), and CT vs. CC model (OR = 1.35, 95% CI = 1.18–1.54, *P* < 0.001), which was associated with an increased risk of CAD significantly. Upregulation of MMP-9 may be involved in the CAD development by multiple approaches, including increased proliferation and migration of VSMCs, remodeling of the injured vascular cells, and enhancing the plaque instability and rupture (that leads to the development of thrombosis), eventuating in myocardial infarction and CAD [72].

In spite of an attempt to perform the most comprehensive meta-analysis of the *MMP9* gene SNPs and the risk of CAD, a number of limitations and caveats of this meta-analysis study should be taken into consideration. First, the number of studies and sample size for MMP-9 (R279Q), MMP-9 (P574R), and MMP-9 (R668Q) polymorphisms in this meta-analysis was relatively small to conclude a valid report of the association of these SNPs and CAD risk. Second, we searched for the articles published in only the English language and a number of potential studies might be omitted. Third, this meta-analysis was based on a crude analysis of the genetic polymorphisms, and the adjusting the analysis by gender, age, and other environmental factors were not implemented. Fourth, we detected some degrees of heterogeneity for the analyzed SNPs, that might stem from difference in genetic stratification and ethnicity, diversity in the environmental factors in different populations, and the detection methods.

## Conclusion

Taken all the evidence into conclusion, this was the most comprehensive evaluation of the four *MMP9* gene SNPs in association with CAD. We reported that MMP-9 (C1562T) SNP conferred a susceptibility risk for CAD in the overall analysis and Asian population. That notwithstanding, other three polymorphisms were not associated with disease risk, probably due to little sample size. Hence, we warrant further studies with respect to evaluation of other *MMP9* gene SNPs in association with CAD. Furthermore, the role of other factors, such as age, gender, environmental contributing factors as well as other *MMP9* gene variations in the analyses ahead will hopefully shed further light on the bona fide association of *MMP9* gene polymorphisms and risk of CAD susceptibility.

## Data Availability

All data that support the conclusions of this manuscript are included within the article.

## Abbreviations

MMPs: Matrix metallo proteinases
CAD: Coronary artery disease
VSMCs: Vascular smooth muscle cells
ECM: Extracellular matrix
CI: Confidence interval
OR: Odds ratio
SNP: Single-nucleotide polymorphism
PRISMA: Preferred reporting items for systematic reviews and meta-analyses
NOS: Newcastle–Ottawa scale
HWE: Hardy–Weinberg equilibrium
